# Arginase deficiency masked by cerebral palsy and coagulopathy—Three varied presentations of Latin American origin

**DOI:** 10.1002/jmd2.12397

**Published:** 2023-10-21

**Authors:** Shelby L. Mills, Paige Roberts, Myla Ashfaq, Kathryn Leal, Hope Northrup, Deborah L. Brown, David Rodriguez‐Buritica, Laura S. Farach

**Affiliations:** ^1^ Department of Pediatrics, Division of Medical Genetics McGovern Medical School at the University of Texas Health Science Center at Houston (UTHealth Houston) and Children's Memorial Hermann Hospital Houston Texas USA; ^2^ College of Osteopathic Medicine of the Pacific‐Northwest Western University of Health Sciences Lebanon Oregon USA; ^3^ Department of Pediatrics, Division of Hematology Oncology McGovern Medical School at the University of Texas Health Science Center at Houston (UTHealth Houston) and Children's Memorial Hermann Hospital Houston Texas USA

**Keywords:** arginase deficiency, ARG1‐D, urea cycle disorders, cerebral palsy, coagulopathy

## Abstract

Arginase deficiency (ARG1‐D) is an autosomal recessive inborn error of metabolism that is often misdiagnosed. Classic presentation of ARG1‐D includes progressive symptoms of spasticity, delayed development, cognitive impairment, protein avoidance, and seizures. Patients who present atypically may evade diagnosis and require a thoughtful diagnostic workup. Here, we discuss three females of Latin American origin with differing clinical presentations, but who all have the same intronic pathogenic variant in *ARG1*. Importantly, we found that each case included elevated coagulopathy on laboratory testing and discussed one case in particular with manifestation of bleeding. When diagnosed early, treatment is favorable and can prevent progressive decline. While many states have added ARG1‐D to their expanded newborn screening panels, still many states and countries do not screen for ARG1‐D, and it can be missed in a healthy newborn. We aim to bring awareness to not only the classic presentation as a necessary consideration for otherwise unexplained spastic diplegia but also to the varied presentations of ARG1‐D.

## 1 INTRODUCTION

Arginase deficiency (ARG1‐D) is a rare genetic inborn error of metabolism (IEM). It is estimated to occur in approximately 1 in 350 000–1 000 000 births,^1^ however, its true incidence is unknown as it often remains underdiagnosed. ARG1‐D is a urea cycle disorder (UCD) caused by biallelic pathogenic variants in the *ARG1* gene, that codes for arginase, an enzyme used in the last step of the urea cycle in the hydrolysis of arginine to urea and ornithine.^2^, ^3^ Pathogenic loss‐of‐function variants in *ARG1* lead to a deficiency in arginase resulting in a buildup of L‐arginine and other toxic metabolites. In contrast to other UCDs, ARG1‐D is distinguished by less frequent and less severe episodes of hyperammonemia that present with chronic toxic effects rather than acute decompensation.^1^

If not identified via newborn screening (NBS), the disease typically presents in early childhood around 3 years of age.^1^ The clinical presentation of ARG1‐D is variable and includes elevated levels of plasma arginine, progressive spasticity, abnormal gait, loss of ambulation, developmental delay, intellectual disability, failure to thrive (FTT), incontinence, and seizures. Spastic diplegia, which typically presents in early childhood, can sometimes be misdiagnosed as cerebral palsy (CP) or hereditary spastic paraplegia (HSP).^4^ Due to the variable presentation and slow progressive nature of ARG1‐D, it is difficult to diagnose and a high degree of clinical suspicion is necessary to catch the disease early on. However, timely diagnosis is critical as treatment via low‐protein diets and nitrogen scavenging agents can be quite effective when offered prior to excessive hyperammonemia and CNS toxicity.^5^

We aim to add to the literature on ARG1‐D by demonstrating the contrasting presentations of three diverse patients with ARG1‐D: one with a classic presentation and two with atypical presentations. These cases expand known clinical variability and raise larger concerns about detection and testing strategies in various populations.

## 2 MATERIALS AND METHODS

### 2.1 Editorial policies and ethical considerations

The study was exempt from IRB approval based on the format of the research. Our patients gave informed consent to have information reported in this study. Existing patients in the literature were reported to have informed consent at the time of the study.

#### 2.1.1 Patient 1: Classic presentation

A 5‐year‐9‐month‐old El Salvadorian female presented to Medical Genetics after referral from Orthopedics for evaluation of weakness, balance issues, and gait abnormalities including increased falling and dragging her feet while walking starting at age 3. She was born in El Salvador to a 29‐year‐old G2 P1 mother and 29‐year‐old father. Pregnancy was complicated by a maternal chikungunya infection at 6 months gestation and she was born full‐term without complication. She was healthy with most developmental milestones achieved at typical times. She had speech delay with vocabulary of 6–10 words at age 3 years. At that time, they noted cognitive decline with difficulty speaking and loss of previously known words. She also had limited activities of daily living including trouble with toileting and dressing herself due to imbalance. She avoided meat and eggs in her typical diet. Family history was unremarkable, with a healthy full sibling.

Physical examination demonstrated difficulty with cooperation and verbal expression as well as a small, frail appearance for her age. Her weight was in the 25–50th percentile while her length and head circumference were at the 3rd percentile. She had protuberant, low‐set ears. She was hypertonic in the gastrocnemius and adductor muscles. She had a broad circumduction gait with bilateral foot drop and minimal knee flexion while walking, sustained clonus at the ankle, and hyperreflexia at the patella bilaterally.

Due to the concern of an underlying genetic etiology, clinical exome sequencing was performed and revealed a paternally inherited pathogenic splicing variant of *ARG1* c.466‐1G>C, which is reported by HGMD Professional 2020.3 to disrupt the highly conserved acceptor splice site. She also had a maternally inherited *ARG1* mutation with a 32 bp deletion and 21 bp insertion (c.314_345delinsATGCAAGCATCAGGATGACT). While the genetic report classified this mutation as a VUS, its pathogenicity can be deduced due to its causation of disease and inherently large mutation.

She had elevated arginine at 559 μmol/L (41–114 μmol/L) on plasma amino acids (PAA) confirming the ARG1‐D diagnosis. Other essential amino acids were low, indicative of protein‐avoidance. She also had borderline elevated guanidinoacetate (GAA) of 1333 (21–1324 nmol/mL), elevated AST of 133 U/L (29–39 U/L), ALT of 289 U/L (8–24 U/L), and ALP of 404 U/L (117–311 U/L) suggestive of liver dysfunction. She also had an elevated prothrombin time (PT) of 14.5 s (9.0–11.5 s) and an international normalized ratio (INR) of 1.5 (0.9–1.1).

When these results were disclosed, the patient was age 6 years old and had continued progression of her symptoms. Her weakness and hypertonia had worsened, particularly in the knees and ankles and she required a walker when ambulating. She also reported new onset fecal incontinence.

Once ARG1‐D was confirmed, she was guided by a metabolic dietician and started on a low‐protein diet including essential amino acid medical food; she was decreased from 1.3 g/kg/day at baseline to 1 g/kg/day 40% intact protein and 60% essential amino medical food. She was also treated with a nitrogen scavenging medication, glycerol phenylbutyrate 4.5 mL/m^2^/day divided TID. After 2 weeks of strict adherence to the low‐protein diet and beginning medication, she had a pronounced reduction in arginine levels from 566 to 381 μmol/L. At her 3 month follow‐up visit, she had improvement in speech, school performance, and bowel/bladder control. There were no improvements in gross motor activities. She had reported several episodes of seizure‐like activity in a day, that then resolved, and is pending neurology follow‐up.

#### 2.1.2 Patient 2: ARG1‐D accompanied by true cerebral palsy

A 4‐year‐old Honduran female was originally seen by a Medical Genetics inpatient when she was found to have hyperammonemia after presenting with a two‐month history of increased frequency of seizures and a two‐week history of decreased oral intake and vomiting. She was born in Honduras to a G2 P1 25‐year‐old mother and 33‐year‐old father with pregnancy complicated by two bouts of dengue fever. She was born full‐term with typical resuscitation. At 2 months, she had normal development with the gain of head control but then acquired viral meningitis with infarcts to the major cerebral artery demonstrable on magnetic resonance imaging (MRI) performed during the hyperammonemic episode. Subsequently, she lost head control, developed spastic quadriplegia, and seizures and was diagnosed with cerebral palsy. She was also nonverbal and had a history of FTT. Family history was noncontributory. She has two healthy siblings.

On physical exam, she measured below the 3rd percentile for both weight and height and in the 50th percentile for a 2.5‐year‐old. Her head circumference was well below the 3rd percentile measuring in the 50th percentile for a 12.5‐month‐old. She appeared generally thin and malnourished, globally delayed, and in mild distress. She was microcephalic with a low anterior hairline. She was wheelchair dependent. She had opisthotonic posturing with extended extremities and contractures, scoliosis, a sacral dimple, and overlapping toes on the left foot. Her neurologic exam revealed hypertonicity throughout, hyperreflexia, and no significant muscle strength against counterforce greater than gravity. She was nonverbal and unable to follow commands.

Initial laboratory workup was positive for elevated liver enzymes: AST of 204 U/L (0–65 U/L), ALT of 154 U/L (0–37 U/L), and ALP of 524 U/L (142–336 U/L). She also had elevated PT of 16 s (14.1 s) and INR 1.31 (0.85–1.17). She had repeated hyperammonemia of 261, 145, and 126 μmol/L (<45 μmol/L), and urinalysis demonstrating urinary tract infection. PAA demonstrated significantly elevated arginine at 4x the upper limit of normal (477 μmol/L). Cerebral Palsy Spectrum Disorder gene panel showed homozygous pathogenic variants of *ARG1* (c.466‐1G>C), interrupting a known splice acceptor. MRI of her brain showed extensive encephalomalacia from prior bilateral middle cerebral artery infarcts. Pelvis X‐ray showed a bilateral hip dislocation.

The hyperammonemia was treated and resolved with the initiation of intravenous fluids containing 10% dextrose. Afterward, she was started on a low‐protein diet with essential amino acid medical food at 2 g/kg/day of protein at baseline decreased to 0.7 g/kg/day, at a ratio of 40% intact protein and 60% essential amino acid medical food. She was also started on nitrogen scavenging medication sodium phenylbutyrate at a rate of 250 mg/kg/day divided TID. A gastrostomy tube was placed to help with the poor intake.

After 2 years of treatment, the patient is clinically stable. No functional improvement was evident, but the family reports ease of mobility in and out of the wheelchair with less frequent crying. Plasma arginine decreased post‐treatment but remains elevated to (247 μmol/L compared to 670 μmol/L on initial presentation, normal 41–114). Her liver enzymes and PT normalized.

#### 2.1.3 Patient 3: Atypical presentation with clotting abnormalities and no spasticity

An 8‐year‐old El Salvadorian female presented to the hospital with new‐onset seizures and recurrent nosebleeds. Genetics was consulted for suspicion of vitamin K receptor deficiency after prolonged PT was found that did not improve with vitamin K administration. She was born in the United States to a G2 P1 26‐year‐old mother and 29‐year‐old father at term with no known birth or pregnancy complications. She had a normal NBS performed in the state of Texas. Family history was positive for consanguinity, as the maternal and paternal grandmothers were first cousins. She had three healthy siblings. Her past medical history was significant for developmental delay, FTT, and nosebleeds. She had delayed walking at 2 years 4 months and delayed speech at 2 years. Otherwise, she was reported to be generally healthy with no motor deficits, spasticity, or prior seizures.

On physical examination at age 8, her weight and height were less than 3rd percentile (−2 and − 3.7 standard deviations, respectively), and head circumference was at the 25th percentile. She looked younger than stated age but was generally non‐dysmorphic, and had normal tone, reflexes, and neurologic exam.

Imaging of the brain including head CT scan and MRI were unremarkable. An electroencephalogram at initial presentation suggested potential epileptic activity, though no further seizures ensued and no diagnosis of epilepsy was made. She had a normal liver ultrasound and normal skeletal imaging; however, she had delayed bone age.

Labs at presentation included elevated transaminases peaking at 219 U/L for AST (0–65 U/L) and 218 U/L for ALT (0–37 U/L), respectively. Labs also showed an elevated PT of 21.5 s (12.0–14.7 s) and INR of 2.25 (0.85–1.17). She also had low Factor VII, IX, and X with an elevated Factor VIII and vitamin K. She did not respond to vitamin K administration. To evaluate for vitamin K receptor deficiency, along with her other medical problems, exome sequencing was recommended but could not be performed due to insurance issues. Her genetic workup was instead performed over time based on what testing would be covered. ARG1‐D was not considered due to lack of spasticity. In turn, she was not diagnosed with ARG1‐D for 2 years, once Whole Exome Sequencing (WES) was covered by her insurance and completed.

Throughout 2 years of follow‐up with Medical Genetics while attempting to authorize WES, other labs included karyotype, chromosomal microarray (CMA), and gene panels for seizures and glycogen storage disorders which were non‐elucidative. The patient's CMA revealed long contiguous regions of homozygosity in chromosomes 5 and 6 consistent with previously reported consanguinity. Of note, *ARG1* is found on chromosome 6 (OMIM). WES was obtained nearly 2 years after initial presentation and showed a homozygous inheritance of pathogenic variant c.466‐1G>C at *ARG1*. Her definitive diagnosis became ARG1‐D and standard of care was implemented. Baseline PAA profile demonstrated several low essential amino acids on and plasma arginine level 5x the upper limit of normal at 607 μmol/L.

Diet modification included a low‐protein diet with essential amino acid medical food. Her baseline was 0.95 g/kg/day and she is prescribed 0.95 g/kg/day at a ratio of 50% intact protein and 50% essential amino acid medical food, however, her actual intake is approximately 0.92 g/kg at a ratio of 75% intact protein and 25% medical food. Nitrogen scavenger medication glycerol phenylbutyrate was also initiated at 6 mL/m^2^/day divided TID. Unfortunately, despite treatment adjustments, her arginine has been difficult to control due to poor acceptance of medical food and continued protein avoidance, resulting in catabolism. During the first several months on a modified diet and prescription nitrogen scavenger treatment, her arginine decreased to mid‐300 s, but her essential amino acids also remained low despite modifications to treatment. Her most recent arginine levels after approximately a year and a half of treatment remain in the upper 400 s to lower 500 s.

While arginine remains elevated, the patient's clinical presentation has improved significantly over 1.5 years of treatment. Food avoidance and vomiting lessened, improving her quality of life, and her oral intake and growth increased. Her height has improved +0.5 z‐scores. No further hospitalizations or seizures were noted. Cognitive impairment remained, though post‐treatment the impairment did not worsen. She has continued to have no physical impairments.

## 3 DISCUSSION

The three highlighted patients represent contrasting phenotypic presentations in an underrepresented patient population, ranging from classical to coagulation abnormalities without spasticity. Our first two patients demonstrate a common pitfall of ARG1‐D phenotypically appearing as CP. These cases highlight the importance of a thorough workup of spastic diplegia phenotypes, specifically the implementation of ARG1‐D screening, and consideration of metabolic testing in atypical presentations.

Diagnosis can be difficult in ARG1‐D, especially in the setting of overlapping and atypical phenotypes. PAA labs are an important screening tool for patients presenting with otherwise unexplained CP. All three of our patients presented with arginine levels 4x or greater than the upper limit of normal, though any increase 3x normal should raise suspicion for ARG1‐D.^1^ Low essential amino acids may help to quantify protein avoidance and was seen in all three patients. Plasma ammonium levels can also be helpful in screening, though hyperammonemia is intermittent in comparison to other UCDs.^6^

When clinical and biochemical features suggest ARG1‐D, genetic testing can be used as confirmation. ARG1‐D displays autosomal recessive inheritance, thus biallelic variants are required for diagnosis. As is expected in enzymatic deficiencies, affected ARG1‐D individuals are found to have a genotype–phenotype correlation such that a decreased amount of residual enzyme results in a more severe phenotype and neonatal onset. Many nonsense mutations are thus considered severe,^6^ the severity of other variants depends on residual enzyme activity. There are 66 reported pathogenic variants, 10 of which are splice site variants.^7^ While clearly, not all nonsense mutations lead to earlier onset, observation of our patients’ variants revealed two were homozygous for the pathogenic intronic variant, c.466‐1G>C. The other patient inherited compound heterozygous variants: the same intronic variant at c.466‐1G>C as well as a maternally inherited 32 bp deletion and 21 bp insertion (c.314_345delinsATGCAAGCATCAGGATGACT). The protein affected with c.466‐1G>C is suspected to be pathogenic.^8^ Previous intronic variants have been described to cause aberrant splicing and result in an exon‐splicing‐enhancer mutation.^9^ This aberrant splicing should be further studied, as it could explain residual enzyme concentrations and correlate with phenotype. Our patients were diagnosed via trio WES or panel testing, and two cases reported distant consanguinity or being from smaller areas of a country.

The first case presented with ARG1‐D in a classic manner, but the second and third cases highlight the clinical variability of manifestations and hurdles in diagnosis. The second patient case included a CP‐like picture that appears to be from viral meningitis in conjunction with ARG1‐D. Her initial diagnosis of ARG1‐D was not evident and she had a typical birth without a traumatic birth event. A case of meningitis subsequently caused multiple brain infarcts evident on MRI. Without records from Honduras, it is impossible to know the causation of her clinical picture; and if hyperammonemia secondary to viral illness played a role in brain damage. The patient did have minor clinical improvements on diet, and plasma arginine and PT lowered. Interestingly, this patient suffered from both a known diagnosis of CP caused by brain assault from meningitis, but also a later‐discovered *ARG1* variant confirming diagnosis of ARG1‐D. While often ARG1‐D can be misinterpreted as CP, it is therefore possible that the two exist concurrently. It is also likely that the preceding CP diagnosis delayed the discovery of ARG1‐D. Importantly, her vomiting, food avoidance (especially protein), and hyperammonemia helped suggest a UCD; while spasticity narrowed to ARG1‐D specifically. PAA lab testing was key in leading to the diagnosis of this patient.

The third patient presented many unusual phenotypes with both seizures and bleeding dysfunction. Interestingly, she did not have spasticity; but did have a history of intellectual disability and slow growth velocity, which are all associated with ARG1‐D. Although seizures are said to occur in approximately half of patients with ARG1‐D, often without hyperammonemia,^6^; these are not thought of as a typical presenting indication. Though the mechanisms of neural toxicity and subsequent spasticity are relatively unclear in ARG1‐D, it is suggested that guanidino compounds play a role. Arginine's structure containing such guanidino groups facilitates the first step of creatine synthesis via the formation of GAA.^10^ GAA is a known epileptogenic compound,^2^ supporting the observed brain and neuromuscular toxicity in patients with elevated arginine. The third patient also presented with the atypical presentation of increased bleeding and abnormal coagulation factors as the primary concern. Prolonged coagulation is considered a clinical sign of ARG1‐D^1^, ^6^ and has been reported by others.^6^, ^11^, ^12^ Given the role of the liver as the main site in coagulation factor synthesis^13^, coagulopathy in ARG1‐D patients is reasonable, though the literature on the mechanism is not well‐established. Recent studies have shown that liver transplantation, even in the absence of hyperammonemia, can normalize coagulopathy in ARG1‐D patients, showing the key role exists in the liver.^11^ In all three cases, elevated PT/INR was observed, and should be considered an ARG1‐D presenting symptom.

All three patients benefited from low‐protein diets demonstrated as decreased arginine and clinical improvement. All had improved lab values and clinical improvement of symptoms at varying degrees, namely with the prevention of hyperammonemia. Additionally, oral nitrogen scavenging medications provided an alternative disposal pathway for nitrogen in amino acid breakdown. Arginine is rich in guanidino nitrogen bases, making nitrogen‐scavenging medications helpful. While the standard of care has shown improved prognoses for patients with ARG1‐D, there remains room for improvement in treatment. Clinical trials for other treatment options, such as enzyme replacement therapy, are underway for this reason. These patients highlight that treatment does have a clinical impact on patients and early diagnosis is the key to initiating treatment prior to hyperammonemia and irreversible damage.

Moreover, all three cases emphasize the importance of NBS. Two of our patients were born outside of the US, and another was born prior to ARG1‐D being included on the Texas NBS. As such, none of our patients received NBS that included analytes for ARG1‐D, and thus, diagnoses were missed until well into childhood. As of 2022, 33 United States/territories have included ARG1‐D on NBS,^14^ making state and country of birth key in diagnosis. ARG1‐D is an IEM that can be reasonably treated with diet modification and nitrogen scavengers, and as such its addition to NBS is one key feature in patients’ prognosis and ending the diagnostic odyssey, along with physician awareness and care.

Interestingly, all our patients presented with Central American ancestry. Two were born in other countries, El Salvador and Honduras, and the third was born in Texas to parents who emigrated from El Salvador. They all share the intronic pathogenic variant, c.466‐1G>C. Diez‐Fernandez additionally reports this variant in their cohort study and cites Hispanic and Guatemalan heritage.^7^ There could be a founder effect for this variant or ARG1‐D in Central America, specifically in the indigenous Mayan population of these bordering countries. This highlights the continued need not only for extensive NBS in America but in countries with high indices of consanguinity and founder variants in ARG1‐D. Further studies could look into population diagnosis and variant analysis in these countries to further support the need for NBS.

In summary, prompt diagnosis of ARG1‐D, even in atypical presentations, is paramount in an effort to reduce arginine toxicity with effective treatment methods. Untreated ARG1‐D leads to toxic metabolic byproducts, with subsequent GDD and neuromuscular problems. It is important to remember ARG1‐D as a differential when spastic diplegia, seizures, intellectual disability, FTT, and GDD are present. Otherwise, unexplained coagulopathy may also be caused by ARG1‐D. Prognosis is best when caught early, ideally on an NBS, and additionally necessary for special populations in detecting patients before irreversible damage occurs. Further studies could examine larger cohorts of patients, particularly those with Central American heritage and atypical presentations. Based on the known treatment, positive outcomes, and variability, ARG1‐D is a key diagnosis to garner pediatric clinical improvements and further diet modifications and treatments could improve outcomes for this rare disorder.

## FUNDING INFORMATION

None.

## CONFLICT OF INTEREST STATEMENT

Dr. Farach has worked as a consultant for Aeglea BioTherapeutics in the past. Shelby L. Mills, Paige Roberts, Myla Ashfaq, Kathryn Leal, Hope Northrup, Deborah L. Brown, and David Rodriguez‐Buritica declare that they have no conflicts of interest to disclose.

## ETHICS STATEMENT

All procedures followed were in accordance with the ethical standards of the responsible committee on human experimentation (institutional and national) and with the Helsinki Declaration of 1975, as revised in 2000 (5).

## INFORMED CONSENT

Informed consent was obtained from all patients for being included in the study. Additional informed consent was obtained from all patients for which identifying information is included in this article.

## Data Availability

The data that support the findings of this study are openly available in OMIM at https://www.omim.org, reference number 20700, and at ClinVar at https://www.ncbi.nlm.nih.gov reference number 419876.
